# Therapeutic Potential of HGF-Expressing Human Umbilical Cord Mesenchymal Stem Cells in Mice with Acute Liver Failure

**DOI:** 10.1155/2016/5452487

**Published:** 2016-02-28

**Authors:** Yunxia Tang, Qiongshu Li, Fanwei Meng, Xingyu Huang, Chan Li, Xin Zhou, Xiaoping Zeng, Yixin He, Jia Liu, Xiang Hu, Ji-Fan Hu, Tao Li

**Affiliations:** ^1^Shenzhen Beike Cell Engineering Research Institute, Yuanxing Science and Technology Building, Nanshan, Shenzhen 518057, China; ^2^Stem Cell and Cancer Center, First Hospital, Jilin University, Changchun 130012, China; ^3^Stanford University Medical School, Palo Alto Veterans Institute for Research, Palo Alto, CA 94304, USA

## Abstract

Human umbilical cord-derived mesenchymal stem cells (UCMSCs) are particularly attractive cells for cellular and gene therapy in acute liver failure (ALF). However, the efficacy of this cell therapy in animal studies needs to be significantly improved before it can be translated into clinics. In this study, we investigated the therapeutic potential of UCMSCs that overexpress hepatocyte growth factor (HGF) in an acetaminophen-induced acute liver failure mouse model. We found that the HGF-UCMSC cell therapy protected animals from acute liver failure by reducing liver damage and prolonging animal survival. The therapeutic effect of HGF-UCMSCs was associated with the increment in serum glutathione (GSH) and hepatic enzymes that maintain redox homeostasis, including *γ*-glutamylcysteine synthetase (*γ*-GCS), superoxide dismutase (SOD), and catalase (CAT). Immunohistochemical staining confirmed that HGF-UCMSCs were mobilized to the injured areas of the liver. Additionally, HGF-UCMSCs modulated apoptosis by upregulating the antiapoptotic Bcl2 and downregulating proapoptotic genes, including Bax and TNF*α*. Taken together, these data suggest that ectopic expression of HGF in UCMSCs protects animals from acetaminophen-induced acute liver failure through antiapoptosis and antioxidation mechanisms.

## 1. Introduction

Acute liver failure (ALF), a severe liver damage caused by a variety of factors, is characterized by serious liver dysfunction of synthesis, detoxification, excretion, and biotransformation. Epidemiological data have shown that therapy-associated or suicide-driven overdosage of acetaminophen (APAP) is the most common etiology of ALF in developed countries [1, 2]. The mechanism of the APAP-induced ALF is related to oxidative stress in liver cells, resulting in the death of a large number of liver cells. Liver transplantation is the most commonly used therapy but has significant limitations due to organ rejection, lack of donors, and high cost [3–5].

Recently, cell-based therapies have focused on the use of mesenchymal stem cells (MSC) for liver regeneration [6], including those MSCs from human bone marrow [7, 8], umbilical cord blood [9], fetal liver [10, 11], or adipose tissue [1, 12–14]. These MSC recipes have been approved to induce host liver recovery and stimulate endogenous regeneration programs [2, 3, 12, 14, 15]. However, the efficacy of these MSC therapies in animal studies still needs to be significantly improved before they can be translated into clinics.

Hepatocyte growth factor (HGF), a potent hepatic mitogen, has multiple physiological and biochemical functions. HGF improves DNA synthesis and acts as an antioxidant factor by decreasing oxidative stress in the liver [16]. In addition, HGF also participates in regulation of various processes in the liver and has been proved to stimulate liver regeneration against liver failure [17, 18]. The HGF/c-Met signaling pathway has been implicated as a key regulator of the cellular redox homeostasis and oxidative stress. The signaling pathway protects against oxidative stress-induced cellular damage [19].

In this study, we determined whether ectopic expression of the human HGF gene would enhance the therapeutic potential of UCMSCs in APAP-induced ALF mice. We hypothesized that this unique therapeutic approach would combine the regenerative role of UCMSCs with the antioxidation activity of HGF factor. For this, we established an ALF mouse model by lethal dose of APAP and examined the therapeutic potential of the HGF-expressing UCMSCs.

## 2. Materials and Methods

### 2.1. Isolation and Flow Cytometry Phenotyping of UCMSCs

Umbilical cords were collected from delivering full-term infants in hospital after obtaining written parental consent. Isolation of UCMSC was performed as described previously [20, 21]. Briefly, Wharton's Jelly was cut into small pieces, treated with collagenase type 1 (Sigma, MO), and then cultured in DMEM containing 10% fetal calf serum (FCS) and antibiotics at 37°C in a 5% CO_2_ humidified atmosphere. Cells that migrated out from the explants after 5–7 days were collected and expanded. Cells at passage 3 were used for cell transplantation. The protocol was approved by the Human Medical Ethical Review Committee from Shenzhen Beike Cell Engineering Research Institute.

The cultured UCMSCs were characterized by cytometry. Cells were trypsinized and suspended at a concentration of 1 × 10^6^ cells/mL in phosphate-buffered saline (PBS) containing 0.1% BSA. Cells were incubated at 4°C with antibodies against MSCs markers (CD90, CD73, and CD105), hematopoietic cell markers (CD45, CD34, CD14, and CD19), and receptors for extracellular matrix (CD29, CD44) and major histocompatibility (HLA-DR) (all from BD Biosciences). After 30 minutes, cells were washed and suspended in 300 *μ*L PBS. Flow cytometry was performed using FACSAria III cell 4 Sorter (BD Biosciences, CA). The experiment was repeated three times, and the results are representative of three independent experiments.

### 2.2. Cloning of Hepatocyte Growth Factor (HGF)

The following PCR primers were used to amplify the human HGF cDNA: HGFNheI-F: 5′-TGCTAGCGCCACCATGTGGGTGACCAAACTCCTGCCAGC-3′ and HGFSalI-R: 5′-AGTCGACCTATGACTGTGGTACCTTATATGTTA-3′. The HGF cDNA was amplified by PCR using pSNAV2.0-hHGF plasmid as the template. PCR products were digested by NheI and SalI restriction enzymes, extracted from agarose gels, and cloned into pDC315 adenoviral vector with T4 DNA ligase.

### 2.3. Generation of HGF-Expressing UCMSCs

Adenoviruses were packaged and produced using the method as previously reported [22, 23]. Adenoviral packaging 293 cells (1 × 10^5^ cells/well) were seeded in 24 well plates in Dulbecco's modified Eagle's medium (DMEM) supplemented with 10% fetal bovine serum (FBS) and incubated at 37°C with 5% CO_2_ overnight. The medium was replaced with Opti-MEM (Invitrogen, CA) before transfection. The cells were transfected at 80%–90% confluence, with the backbone plasmids pBHGlox(delta) E1, 3 Cre and shuttle plasmids pDC315-HGF using Lipofectamine 2000 (Invitrogen, CA) according to the manufacturer's instructions. Six days after transfection, viral plaques appeared and were collected for the production of Ad-HGF.

Human UCMSCs were seeded in T175 flask at a concentration of 10^6^ cells/flask and incubated with growth medium for 24 h. Cells were switched to Dulbecco's modified Eagle medium (DMEM) containing 5 *μ*M HP4 without serum and infected with adenoviral carrying HGF at multiplicity of infection of 50 (MOI = 50). Four hours after viral exposure, medium was changed by normal growth medium and cells were incubated for 48 h. Infected cells were harvested 48 hours later. Flow cytometry was used to characterize the phenotype of HGF-UCMSCs that were transfected with adenoviral HGF.

### 2.4. Immunofluorescence Staining

Immunofluorescence was carried out to study the gene expression in infected cells and control cells. Cultured cells were fixed with freshly prepared 4% paraformaldehyde (PFA) for 10 minutes at RT and permeabilized with 0.1% Triton X-100 for 3–5 minutes on ice. After being blocked with blocking buffer (4% BSA) for 30 min at RT, cells were incubated with rabbit anti-human HGF antibody overnight at 4°C, washed with PBS, and then incubated with the secondary antibodies goat anti-rabbit antibody labeled with PE at RT for 1 h. Slides were washed with PBS. DAPI (4′,6-diamidoino-2-phenylindole, Invitrogen, CA) was used to visualize nuclei. Immunofluorescence was observed under the fluorescent microscope (Observer A1, Zeiss, Germany).

### 2.5. Osteogenic and Adipogenic Differentiation

Cells were seeded at 5 × 10^3^ cells/cm^2^ and were then differentiated using osteogenic and adipogenic differentiation kit (Invitrogen, CA). About 21 to 28 days later, cells were stained with the oil red O or Alizarin Red to detect the presence of neutral lipid vacuoles in differentiated adipocytes and calcium deposition in osteocytes, respectively.

### 2.6. Cell Transplantation into Mice with APAP-Induced Liver Injury

Male BALB/c mice (body weight 18–20 g) were purchased from the Medical Laboratory Animal Center of Guangdong Province, China. Mice were maintained under standard conditions. APAP (Aladdin, Shanghai, China) was dissolved in sterile normal saline (NS) at the concentration of 18.75 mg/mL for the lethal study. An ALF model was generated in 6–8-week-old mice by intraperitoneal administration of a single dose of 800 *μ*L/20 g body weight sterile normal saline (NS) containing 15 *μ*g APAP. After one hour, the mice underwent intravenous tail vein transplantation of HGF-UCMSCs (*n* = 10) or UCMSCs (*n* = 10) at a concentration of 1 × 10^6^ cells per mouse. Control mice were receiving 800 *μ*L/20 g body weight sterile normal saline (NS; *n* = 10). Mouse survival rate was calculated at different time points.

In a second experiment, APAP was prepared as the concentration of 12.5 *μ*g/mL for a sublethal study. In this set of experiments, mice were intraperitoneally injected with 10 *μ*g APAP to make a sublethal ALF model. After cell transplantation, livers were collected for analysis of pathological change and liver functions were assessed.

Animals were euthanized by standard CO_2_ inhalation procedure at the end of the study or if they show the sign of death. All studies were performed under the guidelines and protocols approved by Institutional Animal Ethic Committee of Shenzhen Beike Cell Engineering Research Institute.

### 2.7. Serum Parameter and Antioxidative Enzyme Detection

Blood samples of the sublethal ALF mice were collected at 4 hours, 8 hours, and 24 hours after APAP injected. Blood samples were centrifuged at 3000x rpm, and serum was collected to determine the activities of aspartate aminotransferase (AST), alanine aminotransferase (ALT), and lactate dehydrogenase (LDH). Serum levels of AST, ALT, and LDH in mouse blood were measured with an automated biochemical analyzer (Mindray, Shenzhen, China). One week after transplantation, mice were sacrificed with CO_2_. Parts of the livers were weighed and liver homogenate was made with tissue homogenate machine (IKA). Protein concentration of the liver homogenate was measured with automated biochemical analyzer. The activities of superoxide dismutase (SOD), catalase (CAT), *γ*-glutamylcysteine synthetase (*γ*-GCS), and the levels of malondialdehyde (MDA) and glutathione (GSH) in liver tissues were measured with SOD Assay Kit, *γ*-GCS Assay Kit, CAT Assay Kit, GSH Assay Kit, and MDA Assay Kit (Nanjing Jiancheng Bioengineering Institute, China), respectively. The activities of SOD, CAT, and *γ*-GCS were recorded as the relative activity by normalizing protein concentration of the homogenate. Similarly, the levels of MDA and GSH were recorded as the relative level by normalizing over the protein concentration in homogenates.

### 2.8. Histological and Immunohistochemical Examination

At the end of the study, livers of sublethal ALF mice were removed and weighed. About 50 mg of the liver tissue was grinded in TRIZOL and stored at −80°C for real time PCR. The remainder of each liver sample was fixed in 4% paraformaldehyde for 24 hours and processed for histological and immunohistochemical analyses. Fixed liver samples were cut into small pieces (three from each liver), dehydrated, paraffin-embedded, and cut into 5 *μ*m thickness sections. Sections were stained with hematoxylin eosin for pathological observation. The relative necrotic areas were calculated in three fields under a microscope using the following formula: relative necrotic areas = (necrotic area/total area of image) × 100%. Immunohistochemistry stain was conducted using the method as previously described [8, 24]. Rabbit anti-human HGF monoclonal antibody (Santa Cruz Biotechnologies, CA) and rabbit anti-human CD90 monoclonal antibody (BD Biosciences, CA) were used as the primary antibodies. Goat anti-rabbit polyclonal antibody probed with DAB was used as the second antibody.

### 2.9. Quantitative Real-Time PCR

One week after transplantation, total RNA of sublethal ALF mice was prepared using TRIzol (Invitrogen, CA). RNA was treated by DNase I master mix and converted to cDNA by MMLV RT Mix (Invitrogen, CA). Aliquots of cDNA were tested in PCR assay using SYBR Premix Ex Tq II (Takara, Japan) in a Bio-Rad CFX96 real-time system under standard cycling conditions of 2 min at 95°C, followed by 40 cycles of PCR with 20 sec at 95°C, 30 sec at 56°C, and 30 sec at 72°C. Expression of the target genes (P65, TNF*α*, Bcl-2, and Bax) was calculated as the relative value by normalizing over the expression of GAPDH, using the ΔΔCt method [24, 25]. Data were presented as the fold change in gene expression relative to the negative control group (normal mice). Three replicates were performed for every condition and experiment, with each sample assayed in duplicate for each amplicon. The primers used for PCR included the following:

 P65 F: ACAGACCCAGGAGTGTTCACAGA
 P65 R: CATGGACACACCCTGGTTCAG
 TNF*α* F: ATGAGAAGTTCCCAAATGGC
 TNF*α* R: CTCCACTTGGTGGTTTGCTA
 mBcl2-E1F: GCATCTGCACACCTGGATCCAGGAT
 mBcl2-E2R: GAAATCAAACAGAGGTCGCATGCTG
 mBax-E4F: ACCATCATGGGCTGGACACTGGACT
 mGAPDH-F: AGGTCGGTGTGAACGGATTTG
 mGAPDH-R: TGTAGACCATGTAGTTGAGGTCA

### 2.10. Western Blotting Analysis

The cells were lysed in radioimmunoprecipitation assay (RIPA) lysis buffer (Santa Cruz Biotechnology, CA), and the protein content was determined using Bio-Rad protein assay reagent (Bio-Rad, Hercules, CA). Equal amounts of cell lysate protein were separated by 10% SDS-PAGE and transferred to PVDF membranes (Millipore, Billerica, MA, USA). The primary antibodies against HGF, p65, and *β*-actin (1 : 1000) were derived from Santa Cruz Biotechnology, CA. After washing the membranes with Tris-buffered saline containing 0.05% Tween 20 (washing buffer), horseradish peroxidase (HRP) conjugated secondary antibody (1 : 1000; Santa Cruz Biotechnology, CA) was added. After further washing, color was developed using luminol reagent (Santa Cruz Biotechnology, CA), and the HRP activity of the blots was analyzed using a LAS1000 imager (Fuji film, Tokyo, Japan).

### 2.11. Statistical Analysis

Data were presented as the mean ± standard deviation (SD). The one-way analysis of variance (ANOVA) with the Bonferroni correction was used to analyze differences between two groups of serum parameters. Animal survival was analyzed using the Kaplan-Meier log rank method. *P* < 0.05 was considered to be statistically significant. Data were analyzed with SPSS statistical software.

## 3. Results

### 3.1. Ectopic Expression of HGF Does Not Affect the Multipotency of UCMSCs

**Figure 1:**
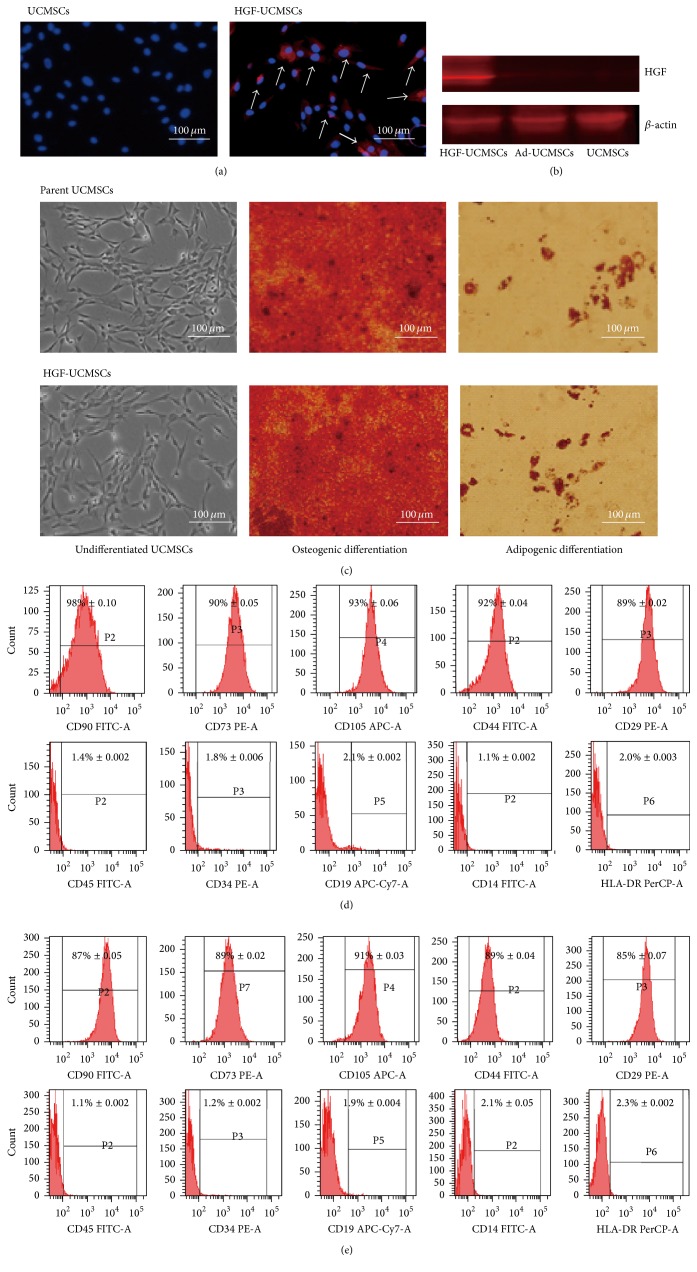
Characterization of human umbilical cord-derived mesenchymal stem cells. (a) Immunofluorescent staining of HGF protein in the liver of HGF-UCMSC treated mice. No HGF was detected in livers from the control group. Cells were HGF-positive (red) two days after HGF adenovirus infection at MOI 50. (b) Western blot of HGF in the liver of treated mice. Ad-UCMSCs: UCMSCs transfected with adenovirus carrying the control vector. (c) Differentiation of human umbilical cord-derived mesenchymal stem cells. Osteogenic differentiation was detected by calcium deposits stained by Alizarin Red. Adipogenic differentiation was detected by oil red O staining (400×). Morphology of UCMSCs and HGF-UCMSCs at day 10 of culture (40x). (d-e) Mesenchymal stem cell marker profile of the parent UCMSCs and HGF-UCMSCs. The data are representative of three independent experiments.

UCMSCs were cultured from human umbilical cord tissues [20]. Adherent UCMSCs began to grow 8–12 days after the initial tissue plating. UCMSCs were infected with HGF adenovirus at a multiplicity of infection of 50. As seen in Figure 1(a), immunofluorescent staining detected the expression of HGF two days after viral transduction (right panel). The parent UCMSCs, however, did not express HGF (left panel). Western blot also validated the expression of HGF in virally transfected UCMSCs (Figure 1(b)).

We then examined the property of HGF-UCMSCs by inducing osteogenic and adipogenic differentiation. After differentiation, we did not observe significant differences between the parent UCMSCs and HGF-UCMSCs in the appearance of intracytoplasmic lipid droplets stained by oil red O (Figure 1(c), right panels) and calcium deposits stained by Alizarin Red (Figure 1(c), middle panels). These data suggest that ectopic expression of HGF does not affect the potential of adipogenic and osteogenic differentiation in UCMSCs.

We further characterized the phenotype of HGF-UCMSCs using flow cytometry 48 hours after viral infection. We found that stem cell markers CD105, CD73, CD90, CD44, and CD29 were equally expressed between the parent and the HGF-expressing UCMSCs (Figures 1(d)-1(e)). Negative markers CD45, CD34, CD14, CD19, and HLA-DR were expressed at very low level in HGF-UCMSCs. Thus, adenoviral expression of HGF does not affect the expression of mesenchymal markers, such as CD90, CD105, and CD73.

### 3.2. HGF-UCMSCs Protect Hepatic Injuries in ALF Mice

To evaluate the therapeutic potential in liver regeneration, HGF-UCMSCs were transplanted into mice with ALF. The ALF model was established in mice by intraperitoneal injection of APAP using a dose that is known to induce oxidative stress, hepatocyte necrosis, extensive vacuolar degeneration, and inflammatory cell infiltration in most of the zones of the parenchyma [26]. To determine the efficacy of HGF-UCMSC in ALF, we performed a titration experiment to determine the appropriate dose of cells and the timing window of cell treatment. We found that 1 × 10^6^ UCMSC could lead to a decrease in the transaminase level. Intravenous transplantation was the most effective method to deliver HGF-UCMSCs. Thus, 1 × 10^6^ HGF-UCMSCs were intravenously transplanted into APAP-injured mice an hour after ALF induction.

**Figure 2:**
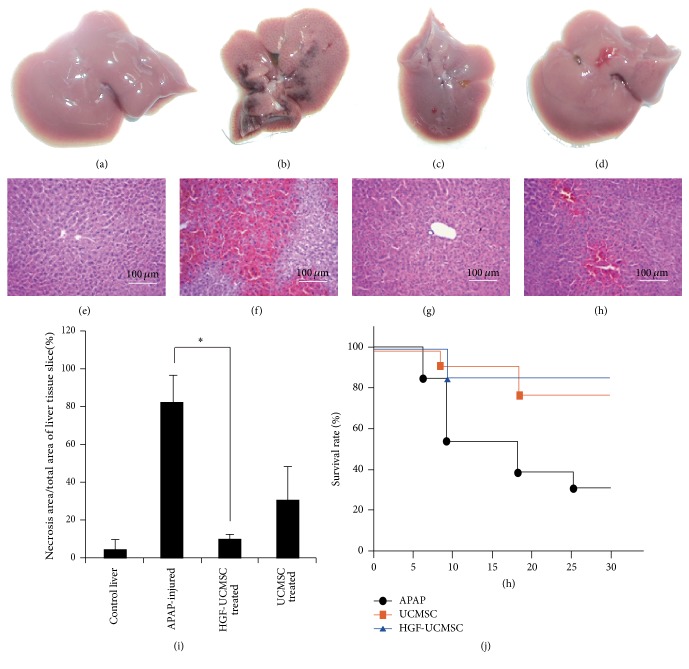
HGF-UCMSC treatment reduces hepatic damage in ALF mice. (a–d) Morphological analysis of the liver. (e–i) HE staining of the necrosis area in liver sections in ALF mice induced by 750 mg/kg acetaminophen. (j) Treatment with HGF-UCMSC and UCMSC significantly prolonged the survival of mice that received a lethal dose of Acetaminophen compared with nontreated animals.

As compared with the control mice (Figure 2(a)), the sublethal APAP treated mice displayed severe internal bleeding and necrosis in the liver (Figure 2(b)). Remarkably, transplantation of HGF-UCMSCs dramatically attenuated the liver damage (Figure 2(c)). H&E staining of liver sections also confirmed the therapeutic potential of HGF-UCMSCs in attenuating the APAP-induced necrosis (Figure 2(g)). Overall, HGF-UCMSCs showed a better therapeutic potency than UCMSCs along in treating ALF (Figures 2(d), 2(h), and 2(i)).

In a separate study, animals were given a lethal dose of APAP to assess the role of HGF-UCMSCs in improving ALF survival. A single dose of 1 × 10^6^ cells was transplanted intravenously into recipient animals. We found that transplantation of HGF-UCMSCs and UCMSCs showed a significantly increased animal survival rate compared to the NS control. ALF mice in the HGF-UCMSC group had a better survival than those in the UCMSC group (85.7% versus 78.6%), although the difference was not statistically different (Figure 2(j)).

### 3.3. Functional Improvement of Injured Liver by HGF-UCMSCs

**Figure 3:**
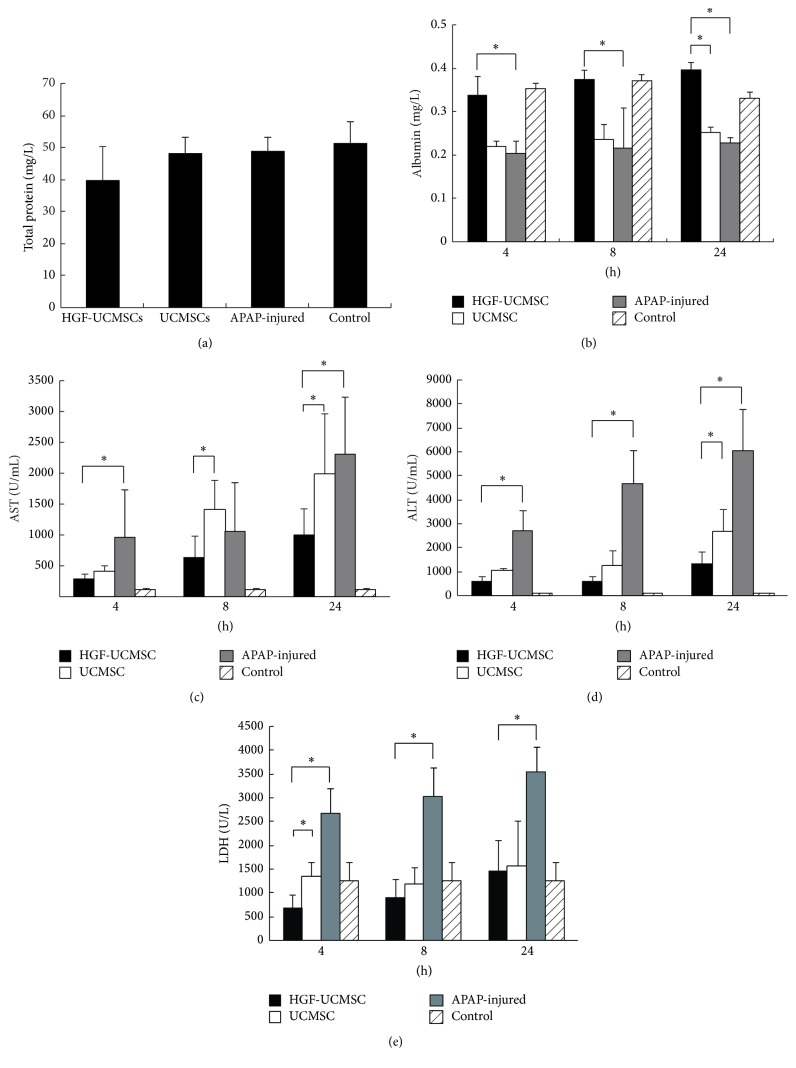
Hepatic function after transplantation of UCMSCs or HGF-UCMSCs. (a) Serum total protein in different groups at time point 24 h. (b) Serum albumin levels in different groups at different time points. (c–e) Biochemical analysis of aspartate aminotransferase (AST), alanine transaminase (ALT), and lactate dehydrogenase in serum. The data are expressed as the means ± SD of three independent experiments. ^*∗*^ *P* < 0.5 compared with the control.

The extent of liver damage in mice induced by sublethal dose of APAP was monitored by serum levels of total hepatic protein and albumin over the 24 h time course of the study. We found that total hepatic protein did not show much difference between different treatment groups (Figure 3(a)). However, serum albumin levels were 1.5-fold higher at 24 h in the mice treated with HGF-UCMSCs than that in the untreated APAP-mouse group and the group treated with UCMSCs (Figure 3(b)).

Hepatic function was also evaluated by the measurement of serum AST, ALT, and LDH. We observed that all of the enzymes were elevated in the ALF mice compared to the control but were decreased at all time points (4 h, 8 h, and 24 h) in the HGF-UCMSC treatment group (*P* < 0.05, Figures 3(c)–3(e)), compared to the untreated APAP-mouse group. Most importantly, the AST and ALT levels at 24 h and the LDH level at 4 h were significantly lower in mice treated with HGF-UCMSC than that in the group treated with UCMSCs.

### 3.4. Migration of HGF-UCMSCs into the Injured Hepatic Zones

**Figure 4:**
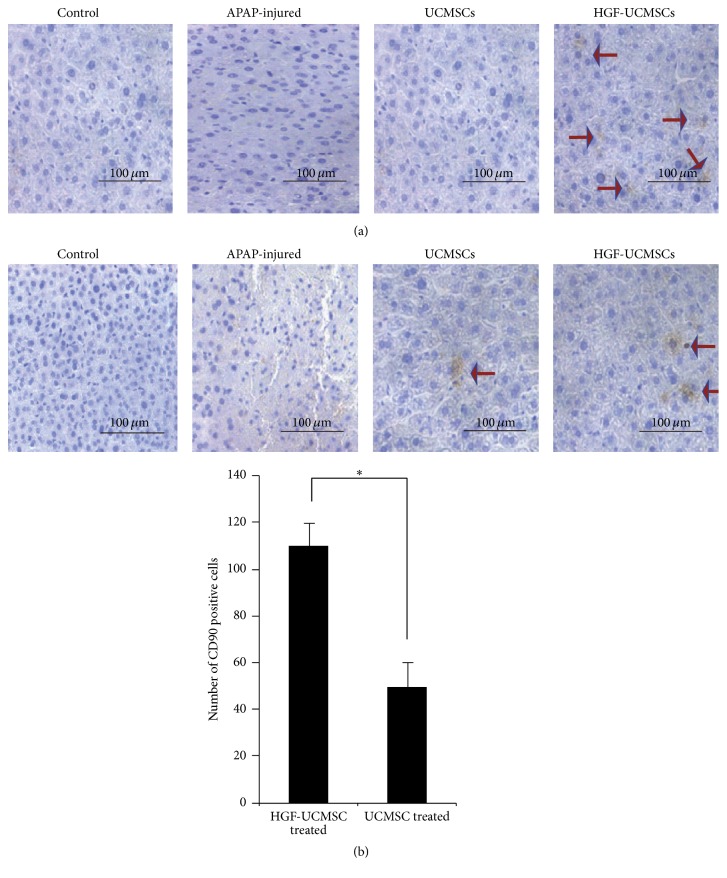
Migration of HGF-UCMSCs into the injured area of the liver. Migrated MSCs in the liver were detected by immunohistochemical staining of HGF (a) and mesenchymal marker CD90 (b).

To delineate mechanisms underlying the protective effect of HGF-UCMSCs in sublethal dose of APAP-induced ALF animals, we tracked the transplanted cells by immunohistochemical staining of human HGF and human CD90 positive cells in the livers of mice on day 7 after transplantation. Using anti-human HGF and anti-human CD90 antibodies, we found that HGF-UCMSCs were detected in the injured liver of mice (Figure 4). These data suggest that the transplanted UCMSCs were able to migrate to the injured area.

### 3.5. HGF-UCMSCs Reduce Oxidative Stress in ALF Mice

**Figure 5:**
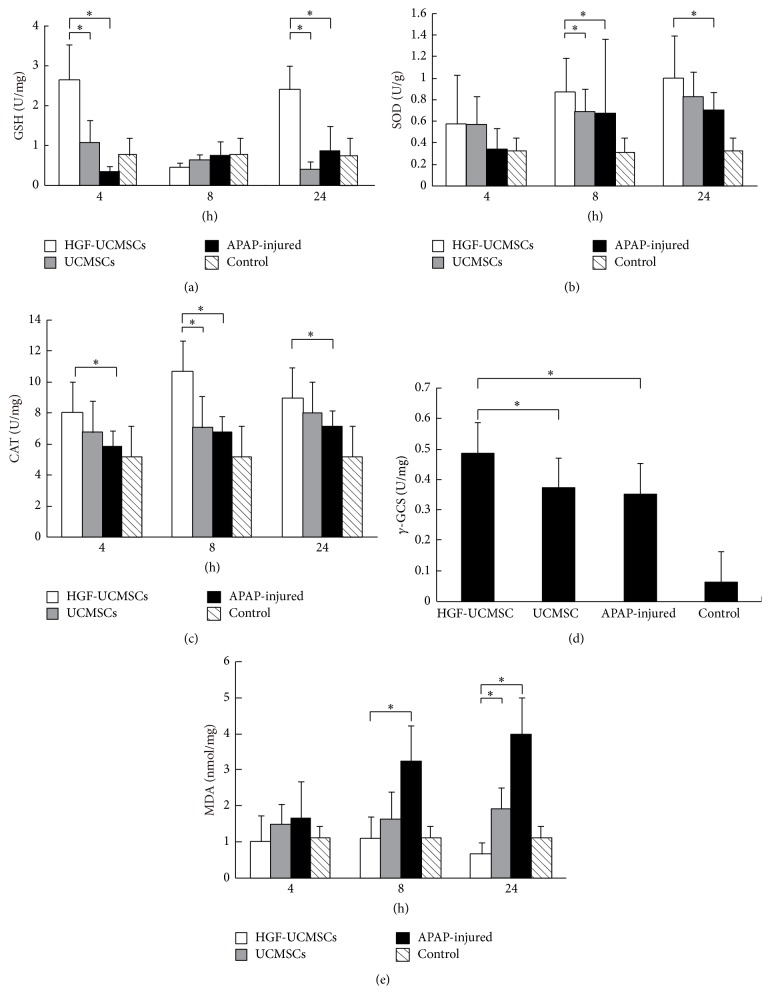
Antioxidant indexes in liver tissue of ALF mice. (a) Level of glutathione. (b) Activity of superoxide dismutase in liver tissue. (c) Activity of catalase in liver tissue. (d) Activity of *γ*-glutamylcysteine synthetase (*γ*-GCS). (e) Level of malondialdehyde (MDA). The data are expressed as the means ± SD of three independent experiments. ^*∗*^ *P* < 0.5 compared with the control.

Overdosage of APAP can induce oxidant stress in hepatocytes, especially GSH exhaustion. The HGF/c-Met signaling pathway is a key regulator of the cellular redox homeostasis and oxidative stress [19]. We thus examined if the treatment of HGF-UCMSCs protected the sublethal dose of APAP-induced ALF animals through a mechanism by modifying the oxidative stress. For this, we examined the level of GSH in the liver. Hepatic GSH was slightly decreased in the APAP-injured mice at 4 h and 8 h compared to the control. Treatment of animals with UCMSCs had no significant effects on the GSH level. However, treatment of HGF-UCMSC significantly improved the GSH level at 4 h and 24 h (Figure 5(a)).

Reactive oxygen species (ROS) are scavenged by cell's antioxidant defense system including superoxide dismutase (SOD), catalase, and glutathione peroxidase. In this system, the SOD enzyme catalyzes the conversion of superoxide anion (O_2_^∙−^) radical into hydrogen peroxide plus molecular oxygen. Glutathione peroxidase and catalase control the balance of oxidants, including ROS, reactive nitrogen species (RNS), and sulphur containing radicals [27–29]. We thus measured these enzymes in our ALF animals. As shown in Figures 5(b)-5(c), the HGF-UCMSC therapy enhanced the activity of both enzymes in APAP-induced ALF animals at 8 h and 24 h.

*γ*-Glutamylcysteine synthetase (*γ*-GCS), a rate-limiting enzyme of GSH synthesis, determines the amount of GSH within the cell. By measuring the activity of hepatic *γ*-GCS in different experiment groups, we found that the HGF-UCMSC treatment increased hepatic *γ*-GCS activity (Figure 5(d))

Exposure to APAP caused the elevation of malondialdehyde (MDA) in the serum. We found that treatment of HGF-UCMSCs significantly suppressed the increment of serum MDA (Figure 5(e)). Notably, HGF-UCMSCs eliminated serum MDA more effectively than UCMSCs at any time points.

### 3.6. HGF-UCMSCs Alleviate Oxidative Stress-Induced Apoptosis

**Figure 6:**
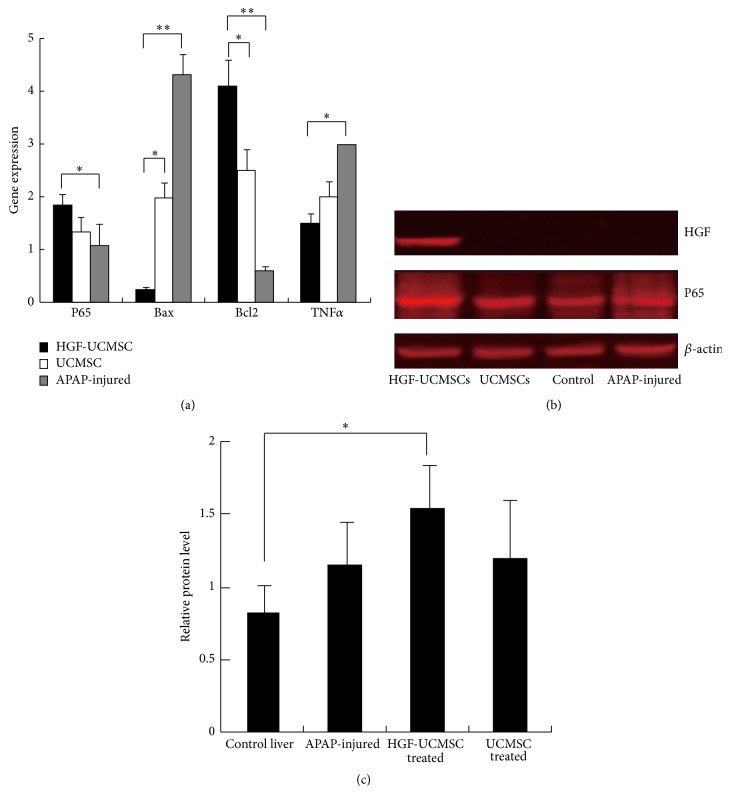
HGF-UCMSCs reduce apoptosis in the liver of ALF mice. (a) Quantitative PCR analysis of mRNA expression of P65, Bcl-2, Bax, and TNF*α* genes in the liver collected from different treatment groups. ^*∗*^ *P* < 0.05; ^*∗∗*^ *P* < 0.01. (b-c) Western blot of HGF and P65 in the liver of ALF mice. The data are expressed as the means ± SD of three independent experiments. ^*∗*^ *P* < 0.5 compared with the control.

Antiapoptosis and regeneration of hepatocytes are critical for preventing the toxicity in sublethal dose of APAP-induced ALF mice. We used quantitative PCR to measure mRNA expression of genes that are associated with cell apoptosis, including Bcl-2, Bax, P65, and TNF*α*. We found that the HGF-UCMSC recipe upregulated the antiapoptotic Bcl-2, but downregulated the proapoptotic Bax and TNF*α* gene at a better efficacy than the USMSC recipe (*P* < 0.01, Figure 6(a)).

Nuclear factor-*κ*B (NF-*κ*B) is a pleiotropic transcription factor that regulates over 200 genes involved in cell growth, apoptosis, tumorigenesis, tumor metastasis, embryonic development, and inflammatory effects. We found that the HGF-UCMSC treatment upregulated the expression of P65 in ALF mice (Figure 6(a)). Similarly, using Western blot we found that the active nuclear P65 of NF-*κ*B was elevated by HGF-UCMSCs (Figure 6(b)). As expected, human HGF was expressed only in the liver of mice from the HGF-UCMSC group. Together, these data suggest that the altered expression of these genes may provide a potential molecular basis for the action of HGF-UCMSCs in the ALF model.

## 4. Discussion

In this study, we for the first time prove the therapeutic potential of the HGF-expressing UCMSCs in mice with ALF. This approach combines the cellular therapy of mesenchymal stem cells with the role of the virally expressed HGF. While UCMSCs aid the regeneration of APAP-induced hepatic necrosis, HGF reduces tissue damage through multiple mechanisms. We demonstrate that systemically administered HGF-UCMSCs protect animals with acute liver failure by alleviating hepatic injuries and prolonging the survival. Notably, overexpression of HGF via adenoviral infection has no detrimental effect on the morphology and multipotent differentiation of UCMSCs.

It is interesting to note that HGF-UCMSCs are capable of improving the survival rate of the injured mice more pronouncedly than the parental UCMSCs. We also detected a better efficacy of HGF-UCMSCs than UCMSCs in improving liver function and pathological changes. Immunohistochemical and HE staining further proved the presence of the homing HGF-UCMSCs within mouse livers, where they may act locally in the injured area to reduce liver necrosis and cell inflammatory infiltration.

HGF, also known as scatter factor, supports diverse cellular functions, including morphogenesis, motility, proliferation, and apoptosis protection [30]. HGF and its receptor c-Met activate signaling pathways that promote cell survival against apoptotic inducers. There are several reports that strongly support the antiapoptotic effects of HGF/c-Met [31–34], primarily by the induction of ant-apoptotic proteins such as Bcl-2, Bcl-XL, or Mcl-1 [35]. In this study, we also found that ALF mice that received the HGF-UCMSC treatment expressed much higher antiapoptotic Bcl2 in the liver than mice that receive the UCMSC treatment. At the same time, the therapy also downregulates the proapoptotic genes, Bax, and TNF*α* (Figure 6(a)). Future studies will be conducted to investigate the detailed mechanisms, particularly apoptosis under different experimental conditions using TUNEL or cleaved-caspase 3 assays.

The role of the HGF/c-Met pathway on oxidative stress protection remains poorly defined. Some published reports indicate that HGF can protect from oxidative damage [36, 37], but the effect of this growth factor on APAP-induced liver damage is practically unexplored. Exposure to APAP causes oxidative stress in liver cells, resulting in a large number of deaths of liver cells. APAP is capable of decomposing and destroying N-acetyl-L-cysteine, which is the necessary synthetic amino acid for glutathione. Cytochrome P450 within the liver cells can eliminate APAP-derived major toxic metabolite and result in the formation of N-acetyl-p-benzoquinoneimine (NAPQI). Sustained formation of NAPQI is conjugated to glutathione and finally causes depletion of glutathione, which then leads to oxidative stress within liver cells [7].

In this study, we demonstrate that HGF-UCMSCs can reduce oxidative stress by increasing cellular glutathione and decreasing MDA content, probably through its ability to enhance the activity of three enzymes (SOD, CAT, and *γ*-GCS) that participate in the maintenance of cellular redox homeostasis. Taken together, our data demonstrate that the HGF-UCMSC therapy combines the unique regenerative role of UCMSCs with the antioxidative activity of human HGF.

MSCs can be directly differentiated into hepatocytic cells under appropriate culture conditions. HGF can induce the expression of liver specific genes, including alpha-fetoprotein, albumin, and cytokeratins, in MSCs through the activation of its receptor c-Met [38–40]. The Mesenchymal and Tissue Stem Cell Committee of the International Society for Cellular Therapy (ISCT) has proposed minimal criteria for human MSCs, including the potential to differentiate into osteoblasts, adipocytes, and chondroblasts* in vitro* [41]. In this preclinical study, we followed the ISCT's criteria and just examined the potential of osteogenic and adipogenic differentiation. The role of HGF in the induction of hepatocytic lineage from UCMSCs will be investigated in future studies. Furthermore, oxidants produced in the cell, including ROS, reactive nitrogen species (RNS), and sulphur containing radicals, are controlled by both pro- and antioxidant enzymes [27, 28]. Future studies are needed to address the changes of oxidants, especially ROS, in our HGF-UCMSC treated ALF model.

In summary, the exposure to various factors, such as drugs, xenobiotics, and viruses, may cause hepatic failure. Findings from this study demonstrate the potential of HGF-expressing UCMSCs in treating ALF. This approach combines the unique regenerative role of UCMSCs with the antioxidation and antiapoptosis properties of human HGF. Our data thus highlight the clinical potential of a valuable two-pronged approach for the treatment of patients with severe liver damage.

## Abbreviations

ALF: Acute liver failure
ALT: Alanine aminotransferase
AST: Aspartate aminotransferase
ELISA: Enzyme linked immunosorbent assay
FCS: Fetal calf serum
GSH: Glutathione
HGF: Hepatocyte growth factor
UCMSC: Human umbilical cord-derived mesenchymal stem cells
MDA: Malondialdehyde
MSC: Mesenchymal stem cells
NS group: Nontransplantation group
NS: Normal saline
PBS: Phosphate-buffered saline
SOD: Superoxide dismutase
CAT: Catalase
SD: Standard deviation
TNF*α*: Tumor necrosis factor-alpha
APAP: Acetaminophen.
